# Aging or chronic stress impairs working memory and modulates GABA and glutamate gene expression in prelimbic cortex

**DOI:** 10.3389/fnagi.2023.1306496

**Published:** 2024-01-08

**Authors:** Hannah M. Gandy, Fiona Hollis, Caesar M. Hernandez, Joseph A. McQuail

**Affiliations:** ^1^Department of Pharmacology, Physiology, and Neuroscience, University of South Carolina School of Medicine, Columbia, SC, United States; ^2^Columbia VA Health Care System, Columbia, SC, United States; ^3^Department of Medicine, Division of Gerontology, Geriatrics, and Palliative Care, The University of Alabama at Birmingham, Birmingham, AL, United States; ^4^Department of Neuroscience, University of Florida, Gainesville, FL, United States

**Keywords:** normal aging, psychogenic stress, prefrontal cortex, glutamate, GABA, glucocorticoids, hypothalamic-pituitary-adrenal axis

## Abstract

The glucocorticoid (GC) hypothesis posits that effects of stress and dysregulated hypothalamic-pituitary-adrenal axis activity accumulate over the lifespan and contribute to impairment of neural function and cognition in advanced aging. The validity of the GC hypothesis is bolstered by a wealth of studies that investigate aging of the hippocampus and decline of associated mnemonic functions. The prefrontal cortex (PFC) mediates working memory which also decreases with age. While the PFC is susceptible to stress and GCs, few studies have formally assessed the application of the GC hypothesis to PFC aging and working memory. Using parallel behavioral and molecular approaches, we compared the effects of normal aging versus chronic variable stress (CVS) on working memory and expression of genes that encode for effectors of glutamate and GABA signaling in male F344 rats. Using an operant delayed match-to-sample test of PFC-dependent working memory, we determined that normal aging and CVS each significantly impaired mnemonic accuracy and reduced the total number of completed trials. We then determined that normal aging increased expression of *Slc6a11*, which encodes for GAT-3 GABA transporter expressed by astrocytes, in the prelimbic (PrL) subregion of the PFC. CVS increased PrL expression of genes associated with glutamatergic synapses: *Grin2b* that encodes the GluN2B subunit of NMDA receptor, *Grm4* that encodes for metabotropic glutamate receptor 4 (mGluR4), and *Plcb1* that encodes for phospholipase C beta 1, an intracellular signaling enzyme that transduces signaling of Group I mGluRs. Beyond the identification of specific genes that were differentially expressed between the PrL in normal aging or CVS, examination of Log2 fold-changes for all expressed glutamate and GABA genes revealed a positive association between molecular phenotypes of aging and CVS in the PrL but no association in the infralimbic subregion. Consistent with predictions of the GC hypothesis, PFC-dependent working memory and PrL glutamate/GABA gene expression demonstrate comparable sensitivity to aging and chronic stress. However, changes in expression of specific genes affiliated with regulation of extracellular GABA in normal aging vs. genes encoding for effectors of glutamatergic signaling during CVS suggest the presence of unique manifestations of imbalanced inhibitory and excitatory signaling in the PFC.

## 1 Introduction

The hypothalamic-pituitary-adrenal (HPA) axis mediates release of glucocorticoids (GC) in response to stressful stimuli (Herman et al., 2003; Smith and Vale, 2006). Age-related dysregulation of the HPA axis prolongs exposure of susceptible corticolimbic neurons to potentially harmful effects of GCs, especially among older individuals with cognitive impairments (Issa et al., 1990; Morano et al., 1994; Yau et al., 1995; Bizon et al., 2001). Indeed, the GC hypothesis of aging stipulates that the accumulating effects of stressful exposures and stress hormones over the lifespan contribute to deleterious neurobiological and cognitive changes that emerge with advanced aging (Sapolsky, 1999; Lupien et al., 2005). While the aforementioned studies focused on mnemonic functions that rely on the hippocampus (HPC), the prefrontal cortex (PFC) is enriched for glucocorticoid receptors (GR; Diorio et al., 1993; Sánchez et al., 2000; Bizon et al., 2001; Chiba et al., 2012; McKlveen et al., 2013), influences HPA axis activity (Herman et al., 1989; Diorio et al., 1993; Radley et al., 2006), and mediates working memory that is highly sensitive to decline in the context of normal aging across rodents, monkeys, and humans (Oscar-Berman and Bonner, 1985; Dunnett et al., 1988; Rapp and Amaral, 1989; Bachevalier et al., 1991; Lamar and Resnick, 2004; Beas et al., 2013). Critically, assumptions derived from associations between HPA axis output and HPC-dependent cognition in aging may not generalize to PFC-dependent working memory as augmented GC secretion during acute stress can strengthen working memory in young adults and associates with preserved working memory in older adults (Yuen et al., 2009; Almela et al., 2014; McQuail et al., 2018). Consequently, further experimentation is needed to empirically affirm, or adjust, predictions of the GC hypothesis of aging in relation to the PFC and working memory.

Working memory depends on persistent firing of PFC pyramidal neurons arising from recurrent excitation via ionotropic NMDA glutamate receptors, balanced with inhibitory GABAergic signaling (Goldman-Rakic, 1995; Rao et al., 2000; Wang et al., 2013). Focused investigation of the aging PFC reveals decline of working memory is mediated by a combination of heightened inhibition, attributed to elevated GABAergic tone (Luebke et al., 2004; Bories et al., 2013; Bañuelos et al., 2014; Carpenter et al., 2016), and diminished excitation, evident as fewer excitatory synaptic connections and lost glutamate receptors (Wenk et al., 1991; Piggott et al., 1992; Magnusson and Cotman, 1993; Magnusson, 1998, 2000; Mitchell and Anderson, 1998; Migani et al., 2000; Magnusson et al., 2007; McQuail et al., 2016, 2021; Hernandez et al., 2018). Stress and GCs are credibly positioned to mediate similar neurobiological changes as chronic stress provokes dendritic regression and spine elimination in PFC neurons via an NMDAR-dependent mechanism while also enhancing inhibitory neurotransmission (Li et al., 2011; Martin and Wellman, 2011; McKlveen et al., 2016). Consequently, there is a compelling rationale to examine the pertinence of the GC hypothesis to signatures of PFC aging by comparing phenotypes of normal aging with those elicited by chronic stress at the levels of working memory and PFC glutamate and GABA. As such, our goals were two-fold. The first goal of this study was to compare patterns of behavioral performance on an operant, delayed match-to-sample test of working memory in rats as a function of chronological age or during chronic stress. The second goal of this study was to use a low-density PCR array approach to simultaneously compare age- or stress-related changes in PFC expression of multiple genes that encode for effectors of glutamate and GABA signaling.

## 2 Materials and methods

### 2.1 Subjects

Male, Fischer 344 (F344) rats were acquired at 4 months (mo.; *n* = 53) or 22 mo. (*n* = 14) of age from the National Institute on Aging’s Rodent Colony housed at Charles River Laboratories. All rats were housed in an AAALAC-accredited vivarium in the McKnight Brain Institute at the University of Florida. The facility was maintained at a consistent 25°C with a 12 h light/dark cycle (lights on at 0700 h) and rats had free access to food and water except for periods of moderate and controlled food deprivation to motivate operant task performance (described below). All animal procedures were approved by the Institutional Animal Care and Use Committee and conformed to the National Institutes of Health’s animal welfare guidelines.

### 2.2 Behavioral methods

#### 2.2.1 Operant testing apparatus, shaping procedures, and delayed match-to-sample task

The operant delayed match-to-sample task (DMTS) was used as a behavioral test of working memory that depends on the rat medial prefrontal cortex (mPFC; Sloan et al., 2006), the rodent homolog of the primate dorsolateral PFC (Uylings et al., 2003). Operant testing apparatus and procedures were identical to those of McQuail et al. (2016, 2018). Operant testing was accomplished using 8 identical operant test chambers (30.5 cm × 25.4 cm × 30.5 cm, Coulbourn Instruments, Whitehall, PA, USA). Each chamber was constructed with metal front and back walls, transparent Plexiglas side walls, and a floor of steel rods (0.4 cm diameter) spaced 1.1 cm apart. The front wall housed a recessed trough that was illuminated by a 1.12 W lamp and connected to a photobeam sensor to detect entries and a food pellet dispenser filled with 45 mg grain-based food pellets (Test Diet, Lab Supply, Northlake TX, USA). Retractable levers were mounted to the left and right of the trough; a 1.12 W cue light was located 3.8 cm above each lever. Each operant chamber was housed inside of a sound-attenuating cubicle that contained a 1.12 W house light and fan mounted on the rear wall. All chambers were connected to a PC running Graphic State 3.01 software (Coulbourn Instruments) to automate behavioral testing and data collection.

Before operant testing, 4 mo. (*n* = 10) or 22 mo. (*n* = 14) were gradually restricted to ∼85% of free-feeding body weight and, on the day before the start of operant shaping, each rat received five food pellets in the home cage to reduce neophobia toward the food reward that was used to positively reinforce operant behaviors. In the first stage of shaping, rats learned to obtain food rewards that were dispensed as 38 deliveries of a single food pellet with an intertrial interval of 100 ± 40 s in a 64-min session. In the second stage of shaping, the left or right lever (counterbalanced across rats) was extended into the chamber and a press resulted in delivery of a single food pellet. After achieving 50 lever presses in a 30-min session, the opposite lever was inserted and reinforced using the same procedure. In the third stage of shaping, rats trained to nose poke into the trough to trigger extension of the left or right lever (counterbalanced between successive pairs of trials) and to press the extended lever to obtain a food reward; rats trained until emitting 80 lever presses in a 30-min session.

Following acquisition of basic operant procedures (nose poke, lever press, and collection of food rewards from the trough), rats were tested on one test session per day wherein they were reinforced to use a “matching” lever pressing rule. During each 40-min test session, the house-light was illuminated throughout except during timeout periods following incorrect responses. The “sample phase” of each trial began with the extension of the left or right lever (randomly selected within each pair of trials) for the rat to press. After pressing, the lever was retracted and the “delay phase” of the trial began. During the delay, rats were required to nose poke into the food trough, the first nose poke after the programmed delay initiated the third and final phase of the trial, the “choice phase.” Because no cues signaled the conclusion of the “delay phase,” rats effectively nose poked continuously until the start of the “choice phase” was signaled by the extension of both levers. Requiring rats to nose poke in the food trough throughout the delay interval also diminishes the development of alternative, “mediating” strategies (e.g., positioning themselves in front of the sample lever during the delay). During the “choice phase,” pressing the same lever that was presented in the “sample phase” was recorded as a “correct” matching response and resulted in retraction of both levers and delivery of a food pellet into the food trough, followed by a 5-s intertrial interval. Pressing the lever that was not presented in the “sample” phase was recorded as an “incorrect” non-matching response and resulted in retraction of both levers and initiation of a 5-s “timeout” when the house light was extinguished, and no food reward was dispensed. Initially, no delay was interposed between the nose poke and the “choice phase” and incorrect choices were followed by a correction trial wherein the sample lever was re-presented on the same side to reduce development of side biases. After reaching 80% correct choices per session for two consecutive sessions, the correction procedure was discontinued and variable, timed delays were inserted between the “sample” and “choice” phases. The sequence of delay intervals was randomized within each block of seven trials; each was presented once within a block. After establishing at least 80% correct responses across two consecutive sessions using a specific set of delays, rats progressed to test with increasingly longer sets of delays (first set of delays: 0, 1, 2, 3, 4, 5, and 6 s; second set of delays: 0, 2, 4, 8, 12, and 16 s; third set of delays: 0, 2, 4, 8, 12, 18, and 24 s). Rats were trained on the third set of delays until reaching stable baseline performance (defined as < 10% variability across five consecutive days of training).

#### 2.2.2 Operant testing during chronic variable stress

Operant testing apparatus and methods to shape behavioral performance were identical to those described for testing of normally aging rats. Following acquisition of stable baseline performance in the DMTS task while testing on the final set of delays, identical numbers (12/treatment) of 4 mo. rats were assigned to unstressed (UNS; control) or chronic variable stress (CVS; experimental) treatment conditions in a manner that counterbalanced for individual differences in performance. Rats assigned to UNS or CVS conditions continued daily testing on the DMTS task, but rats assigned to CVS were exposed to a pseudorandom schedule of specific stressors twice each day after behavioral testing. Stress exposures were conducted in a separate room that was neither the room used for behavioral testing, nor the room used for central housing of home cages. Experimental stressors were (1) 60 min of physical restraint in a Plexiglas cylinder, (2) 15 min of forced swim in 25 cm deep, 25°C water, (3) 7 min of forced swim in 25 cm deep, 15°C water (4) 20 min in an unlined cage filled with 2 cm of 25°C water, (5) 15 min in an unlined cage with cotton ball wetted with coyote urine (Outdoor Maine, Hermon, Maine, USA), and (6) 15 min in an unlined cage with cotton ball wetted with bobcat urine (Outdoor Maine). Following forced swims, rats were thoroughly towel-dried and placed in recovery cages lined with fresh paper towels and a warmed, microwavable heating pad (SnuggleSafe) for at least 30 min. Cotton balls wetted with predator urine were placed in polypropylene tubes that prevented rats from directly contacting the cotton or urine; exposures to predator urine odor were conducted within fume hoods to ensure that odorants did not escape into the ambient air space of the lab. The first daily stressor was delivered shortly after regular operant testing and the second daily stressor was delivered in the afternoon or evening so that the stressor and any subsequent recovery concluded, and rats returned to their home cages, before the start of the dark phase of the light cycle. UNS rats underwent operant testing but were returned to home cages after daily testing and were not exposed to experimental stressors.

#### 2.2.3 Statistical analysis

Data were analyzed using JASP version 0.16.4 (University of Amsterdam, Amsterdam, Netherlands) and α = 0.05 for all comparisons. All data are reported as mean ± standard error; Cohen’s d and 95% confidence intervals are used to report statistically reliable, standardized effect sizes between groups. The total number of correct and incorrect choices after each delay were transformed to the percentage of correct choices and total trials completed.

In the aging cohort, the percentage of correct choices was analyzed using a mixed factors ANOVA testing effects of age (2 levels: young adults or aged) as a between-subjects factor and delay (7 levels: 0, 2, 4, 8, 12, 18, and 24 s) as a repeated measure. The Huynh-Feldt correction was applied, as needed, to correct results where Mauchly’s test of sphericity indicated that the assumption of sphericity was violated. Total trials completed were compared between young adult and aged rats using an independent-samples *t*-test.

In the CVS cohort, the percentage of correct choices (averaged across all delays) and total trials completed were analyzed using a mixed factor ANOVA to test effect of CVS (2 levels: UNS or CVS) in blocks of 7 sessions (4 levels; baseline performance in Pre-CVS Days 1–7, and Post-CVS Days 1–7, 8–14, and 15–21). Significant results were explored within training blocks by means of focused ANOVAs to assess delay (7 levels) as a repeated, within-subjects factor and UNS and CVS treatment as between-subject factors to investigate delay-dependent changes in working memory. Significant effects of CVS on trials completed were explored using independent samples *t*-tests.

### 2.3 Gene expression

#### 2.3.1 mPFC gene expression assays in normally aging rats

After the conclusion of behavioral testing, young and aged rats were restored to *ad libitum* food access and rested in home cages for 2 weeks before euthanasia and brain tissue harvesting for molecular analysis (as in Ianov et al., 2016). The entire brain was removed, frozen in a dry ice-isopentane bath, and stored at −80°C. Frozen brains were equilibrated to −10°C in a cryostat and 350 micron-thick sections were made through the frontal lobe. We collected 1 mm-diameter tissues from the prelimbic (PrL) and infralimbic (IL) subregions of the mPFC using sterile biopsy needles. Tissue punches were ejected into Buffer RLT (PN:74034, Qiagen, Germantown, MD, USA) and immediately homogenized with a hand-held motorized micro-pestle. RNA was isolated using the RNeasy Plus Micro Kit (PN: 74034, Qiagen) according to manufacturer specifications. RNA concentrations were then quantified using a NanoDrop™ One (Thermo Fisher Scientific, Waltham, MA, USA) and RNA integrity was assess using an Agilent^®^ 2100 Bioanalyzer. 50 nanograms of RNA from each sample were reverse transcribed to cDNA via RT2 PreAMP cDNA Synthesis Kit (PN:330451, Qiagen). 5 microliters of cDNA were then amplified using the RT2 PreAMP cDNA Synthesis Kit along with RT^2^ PreAMP cDNA Synthesis Primer Mix for Rat GABA and Glutamate PCR Array (PN: 330241, PBR-152Z, Qiagen) using manufacture’s protocol. Amplified cDNA was then loaded into RT^2^ Profiler™ PCR Array Rat GABA and Glutamate (PN: 330231, PARN-152Z, Qiagen) which were preloaded with 84 primer assays for related genes relevant to GABA and Glutamate. Gene expression was quantified using the Bio-Rad iCycler.

#### 2.3.2 mPFC gene expression assays in chronically stressed rats

A separate cohort of 4 mo. rats (*n* = 9 UNS; *n* = 10 CVS), which were not food restricted or trained on the operant DMTS test, was used to evaluate effects of CVS on mPFC gene expression. The CVS procedure was the same as used in the working memory study and rats were killed approximately 18 h after the final stressor on the 21st day of CVS. Absent behavioral data, we measured change in body weight and adrenal gland weight as weight loss and adrenal hypertrophy are reliable physiological sequelae of chronic stress (Watanabe et al., 1992). Those data were compared between UNS and CVS treatment groups by means of independent-samples *t*-tests. Tissue collection, mPFC microdissection, nucleic acid isolation, and gene expression assays were identical to methods used to process tissue from the normally aging cohort.

#### 2.3.3 Analysis of mPFC gene expression

The Allen Brain Atlas (atlas.brain-map.org; Allen Institute, Seattle, WA, USA) was consulted to identify genes expressed in the rodent cortex (61/89 genes corresponding to assays incorporated into the selected PCR array); non-expressed or lowly expressed genes (Log2 < 0) were excluded from analysis (28/89 genes). *Rplp1* was used as a house-keeping gene to normalize gene expression; *Actb*, *B2m*, *Hprt1*, or *Ldha* were not used for normalization as analysis of raw cycle threshold (Ct) values were determined to differ significantly between groups in either the PrL or IL or were not normally distributed in both regions of all groups. Raw Ct values of genes of interest were normalized to *Rplp1* using the ΔΔCt method. Normalized expression was compared between young and aged rats or UNS and CVS rats by means of independent samples *t*-tests; given the low number of significantly regulated genes detected in this study we did not correct for comparison- or family-wise error rates. To examine global profiles between brain regions and across cohorts, bivariate correlations were implemented using Log2(FC) values of all expressed GABA and Glutamate genes, the strength of association was determined using Pearson’s *r*.

## 3 Results

### 3.1 Aging or chronic variable stress impaired working memory accuracy and reduced trials

**FIGURE 1:**
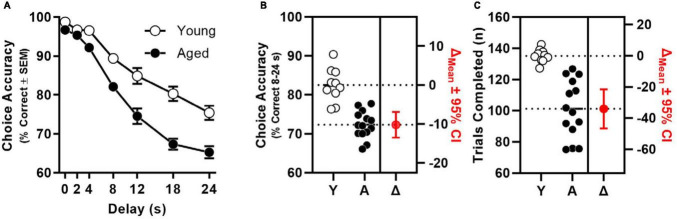
Age-related impairment of mPFC-dependent working memory. **(A)** Choice accuracy (*y*-axis) as a function delay (*x*-axis) of aged (filled circles, *n* = 14) is significantly worse than to young adults (open circles, *n* = 10) in a manner than interacts with delay (age: *p* < 0.001; interaction: *p* < 0.001). **(B)** Choice accuracy (averaged across 8, 12, 18, and 24 s delays; left *y*-axis) of individual young and aged rats; estimation plot of change (Δ) in mean choice accuracy between young and aged (in red; *p* < 0.001; right axis). **(C)** Trials completed (left *y*-axis) by individual young and aged rats; estimation plot of change (Δ) in trials completed between young and aged (in red; *p* < 0.001; right axis).

Consistent with findings of previous studies (McQuail et al., 2016, 2018), age significantly decreased the percentage of correct choices [*F*(1, 22) = 40.095, *p* < 0.001] in a delay-dependent manner [interaction: *F*(4.632, 101.897) = 6.671, *p* < 0.001; Figure 1A]. Indeed, when percentage of correct choices was averaged across 8–24 s delays (i.e., conditions when accuracy of both age groups was < 90%), a large and statistically reliable effect of age was observed [*t*(22) = 6.404, *p* < 0.001; Cohen’s *d* = 2.651; Figure 1B]. The total number of trials completed was also determined to differ reliably between age groups [*t*(14.833) = 6.591, *p* < 0.001; Cohen’s *d* = 2.515; Figure 1C].

**FIGURE 2:**
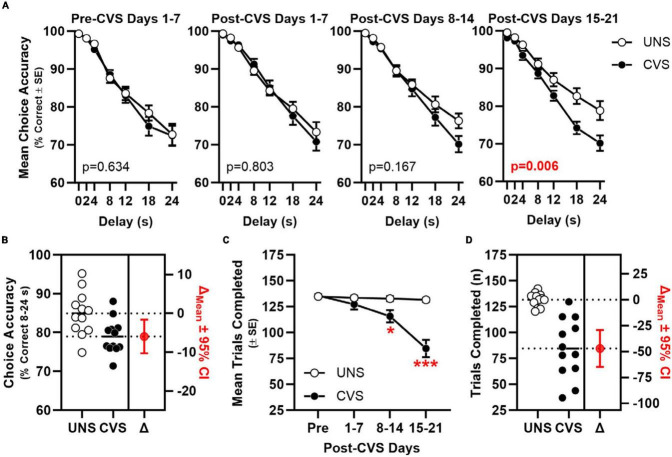
Chronic stress impairs mPFC-dependent working memory. **(A)** Choice accuracy (*y*-axis) plotted as a function delay (*x*-axis) across 7-day blocks (panels) of young adult rats exposed to CVS (filled circles, *n* = 12) or UNS controls (open circles, *n* = 12); choice accuracy of CVS was significantly worse than UNS by Block 4 (after 21–28 days of CVS) in a manner that interacts with delay (stress: *p* = 0.006; interaction: *p* = 0.009) Inset: *p*-value for main effect of CVS. **(B)** Choice accuracy (averaged across 8, 12, 18, and 24 s delays; left *y*-axis) of individual UNS and CVS rats; estimation plot of change (Δ) in mean choice accuracy between UNS and CVS (in red; *p* = 0.009; right axis). **(C)** Mean trials completed (y-axis) plotted as a function of 7-day blocks; CVS-treated rats completed fewer trials compared to UNS controls between after days 8–14 and 15–21. **(D)** Trials completed (left *y*-axis) by individual UNS and CVS rats; estimation plot of change (Δ) in trials completed between UNS and CVS (in red; *p* < 0.001; right axis). *p < 0.05, ***p < 0.001 vs. UNS.

Chronic variable stress (CVS) impaired choice accuracy in a manner that interacted with time [CVS × 7-Day Block interaction: *F*(2.389, 228.544) = 5.747, *p* = 0.003; Figure 2A]. When simple main effects of stress were compared in each block, accuracy differed between UNS and CVS treatment groups in Post-CVS Days 15–21 (*p* = 0.006) but not in Pre-CVS Days 1–7 (baseline; *p* = 0.634), Post-CVS Days 1–7 (*p* = 0.803), or Post-CVS Days 8–14 (*p* = 0.167). An ANOVA focused on choice accuracy in Post-CVS Days 15–21 confirmed the effect of CVS [*F*(1, 22) = 9.325, *p* = 0.006] and further determined an interaction between CVS and delay [*F*(3.697, 81.333) = 3.754, *p* = 0.009]. Choice accuracy averaged across 8–24 s delays in Post-CVS Days 15–21 differed reliably between UNS- and CVS-treated rats and indicated a large effect size [*t*(22) = 2.853, *p* = 0.009; Cohen’s *d* = 1.165; Figure 2B]. The number of trials completed also differed between UNS and CVS-treated rats [*F*(1, 22) = 13.134, *p* = 0.002] in a manner that interacted with block [*F*(1.981, 43.581) = 23.791, *p* < 0.001; Figure 2C]. *Post-hoc t*-tests showed no effect of CVS in Pre-CVS Days 1–7 (*p* = 0.755) or Post-CVS Days 1–7 (*p* = 0.260), but CVS-treated rats completed fewer trials than UNS controls in Post-CVS Days 8–14 (*p* = 0.016; Cohen’s *d* = 1.096) and Post-CVS Days 15–21, (*p* < 0.001; Cohen’s *d* = 2.231; Figure 2D).

**FIGURE 3:**
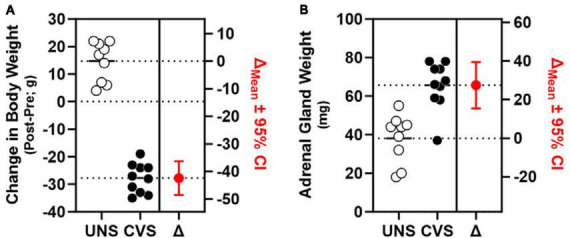
Chronic stress induces weight loss and adrenal hypertrophy. **(A)** CVS-exposed rats (filled circles; *n* = 10) exhibited significant weight loss (left *y*-axis) compared to UNS controls (open circles; *n* = 9) estimation plot of change (Δ) in body weight between UNS and CVS (in red; *p* < 0.001; right *y*-axis). **(B)** Weight of adrenal glands (left *y*-axis) was significantly greater in CVS rats compared to UNS; estimation plot of change (Δ) in adrenal weight between UNS and CVS (in red; *p* < 0.001; right *y*-axis).

In a second cohort of rats that were exposed to CVS, but not concurrently food-restricted or assessed on the DMTS task, efficacy of the CVS regimen was apparent as significant weight loss in CVS-treated rats compared to UNS controls [*t*(17) = 14.64, *p* < 0.001, Cohen’s *d* = 6.726; Figure 3A]. Efficacy of CVS was also evident in greater adrenal weight of CVS-treated rats compared to UNS controls [*t*(17) = −4.812, *p* < 0.001; Cohen’s *d* = −2.211; Figure 3B].

### 3.2 Aging or chronic variable stress increased expression of specific genes and global GABA and glutamate gene expression was correlated between aging and chronic stress in PrL but not IL

**FIGURE 4:**
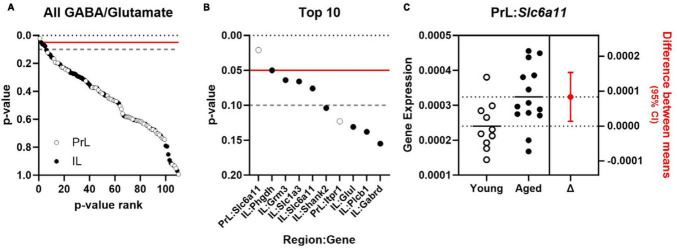
Expression of *Slc6a11* is increased in PrL of normally aging rats. **(A)** *p*-values comparing effect of age of 55 GABA and glutamate genes (*y*-axis) expressed in PrL (open circles) and IL (closed circles) plotted as a function of rank (*x*-axis). **(B)** *p*-values for top 10 ranked GABA and glutamate genes (*y*-axis) in PrL (open circles) and IL (closed circles) plotted as a function of rank (*x*-axis). Horizontal lines in **(A, B)** denote threshold of *p* = 0.05 (solid red) and *p* = 0.1 (dashed gray). **(C)** Expression (left) of *Slc6a11* (*y*-axis) in PrL of young adult (*n* = 10; open circles) or aged rats (*n* = 14; closed circles) and associated estimation plot (right) illustrating the change in mean expression between young adult and aged rats ± 95% CI. Solid and dashed horizontal lines denote mean expression for each group.

**FIGURE 5:**
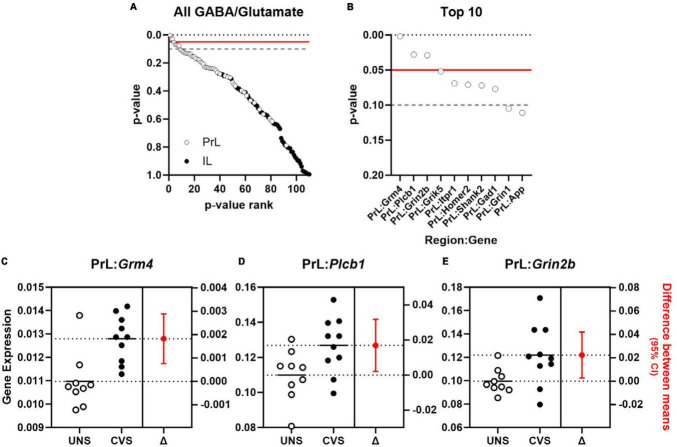
Expression of genes associated with glutamate signaling are increased in PrL of chronically stressed rats. **(A)** *p*-values comparing effect of CVS of 55 GABA and glutamate genes (*y*-axis) expressed in PrL (open circles) and IL (closed circles) plotted as a function of rank (*x*-axis). **(B)** *p*-values for top 10 ranked GABA and glutamate genes (*y*-axis) in PrL (open circles) and IL (closed circles) plotted as a function of rank (*x*-axis). Horizontal lines in **(A, B)** denote threshold of *p* = 0.05 (solid red) and *p* = 0.1 (dashed gray). (**C–E)** Expression (left *y*-axis) of *Grm4* **(C)**, *Plcb1* **(D)**, and *Grin2b* **(E)** in unstressed (UNS; *n* = 9; open circles) or rats exposed to chronic variable stress (CVS; *n* = 10; closed circles) and associated estimation plot (right) illustrating the change in mean expression (right *y*-axis) between UNS and CVS rats ± 95% CI. Solid and dashed horizontal lines denote mean expression for each group.

**FIGURE 6:**
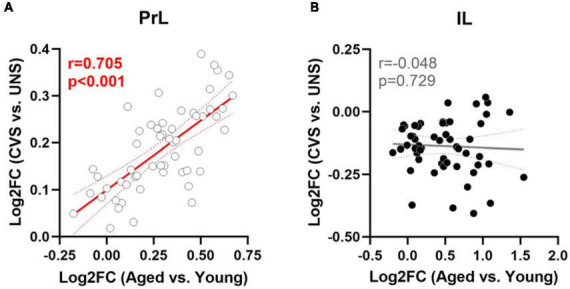
Similar modulation of gene expression between aging and CVS in PrL but not IL. **(A)** Log2FC values of age (*x*-axis) vs. CVS (*y*-axis) of 55 expressed genes in PrL. **(B)** Log2FC values of age (*x*-axis) vs. CVS (*y*-axis) of expressed genes in IL. Lines of best fit and 95% confidence bands are plotted with associated Pearson’s *r* and *p*-values; significant associations are noted in red.

Effects of aging on 55 genes expressed in PrL and IL (Figures 4A, B) determined that expression of *Slc6a11*, which encodes for GABA transporter type 3 (GAT-3), was significantly greater in the PrL of aged rats compared to young adults [*t*(22) = −2.487, *p* = 0.021; Cohen’s *d* = −1.030; Figure 4C] with trends (*p* < 0.1) toward effects of aging on expression of *Phgdh* (phosphoglycerate dehydrogenase), *Grm3* (glutamate metabotropic receptor 3), *Slc1a3* (glutamate-aspartate transporter 1; EAAT1/GLAST), and *Slc6a11* in the IL. Parallel analyses of the same genes differentially expressed between UNS and CVS rats (Figures 5A, B) revealed significantly greater expression of *Grm4* [glutamate metabotropic receptor 4; *t*(17) = −3.622, *p* = 0.002; Cohen’s *d* = −1.664; Figure 5C], *Plcb1* [phospholipase C beta 1; *t*(17) = −2.395, *p* = 0.028; Cohen’s *d* = −1.101; Figure 5D], and *Grin2b* [glutamate ionotropic receptor NMDA type subunit 2B; *t*(17) = −2.392, *p* = 0.029; Cohen’s *d* = −1.099; Figure 5E] in the PrL of CVS relative to UNS. There were similar trends (*p* < 0.1) toward greater expression of *Grik5* (glutamate ionotropic receptor kainate type subunit 5), *Itpr1* (inositol 1,4,5-trisphosphate receptor, type 1), *Homer2* (homer scaffold protein 2), *Shank2* (SH3 and multiple ankyrin repeat domains 2), and *Gad1* (glutamate decarboxylase 1/GAD-67) in PrL of CVS vs. UNS. Using Log2(FC) values of all expressed genes, reflecting direction and size of fold change in gene expression for age or CVS, we determined that the effects of age and CVS on global gene expression were positively correlated in the PrL (*r* = 0.705, *p* < 0.001; Figure 6A) but not correlated in the IL (*r* = −0.048, *p* = 0.729; Figure 6B).

## 4 Discussion

The GC hypothesis of aging implicates accumulating effects of stress and HPA axis dysfunction during later life neural declines. Such changes are thoroughly studied and well-substantiated in the context of HPC, memory loss, and Alzheimer’s disease (Hartmann et al., 1997; Wilson et al., 2005, 2011; Csernansky et al., 2006; Ennis et al., 2017; Majoka and Schimming, 2021). The PFC is also sensitive to aging and dysregulated HPA axis output, but associations between HPA axis activity and cognition vary between PFC- and HPC-dependent forms of cognition (Issa et al., 1990; Morano et al., 1994; Yau et al., 1995; Bizon et al., 2001; Almela et al., 2014; McQuail et al., 2018). This study systematically investigated effects of normal aging or chronic stress in rats at the level of mPFC-dependent working memory and expression of glutamate- and GABA-related genes localized to mPFC subregions to determine the degree to which chronic stress replicates the cognitive and molecular consequences of PFC aging. Broadly, our data show that the GC hypothesis of aging extends beyond the HPC to the PFC, while also highlighting that certain aspects of excitatory or inhibitory signaling may be more susceptible to regulation by aging vs. chronic stress. Normal aging is associated with impaired choice accuracy and fewer trials completed on a self-paced operant test of working memory and similar changes are induced in young adult rats exposed to 21 days of chronic stress. At the level of the mPFC, the profile of effect magnitude and direction for expressed glutamate- and GABA-related mRNAs in aging or chronic stress were reliably and positively correlated in the PrL, suggesting the potential for a cooperative relationship between stress and aging on mPFC gene expression networks. At the level of specific transcripts, differentially expressed genes involved GABA transport in the aged PrL but glutamatergic neurotransmission in the stressed PrL, highlighting potential cell population specificity.

### 4.1 Changes to working memory during stress and in advanced aging

Working memory is a core component of executive functions that are central to normal development, mental health, and personal independence (Diamond, 2013); impaired working memory and executive dysfunction are evident in stress-linked neuropsychiatric disorders (Price and Duman, 2020) and advanced aging (Cohen et al., 2019). The first major finding of our study is that chronic, variable stress delivered to young adult, male rats lowered accuracy on an operant test of mPFC-dependent working memory that is also sensitive to decline in normally aging male rats. This observation agrees with previous findings that chronic variable stress impairs performance on forms of cognition that involve the rodent mPFC, including attentional set-shifting and spatial working memory (Bondi et al., 2008; Anderson et al., 2020). In particular, the T-maze has been instrumental in characterizing spatial working memory deficits induced by sustained exposure of rats to both repeated stressors (Mizoguchi et al., 2000; Hains et al., 2009, 2015; Hinwood et al., 2012; Lee and Goto, 2015) as well as varied and unpredictable stressors (Anderson et al., 2020). While the T-maze is certainly sensitive to mPFC-specific experimental manipulations, it is also critical to recognize that HPC processes contribute to spatial working memory, as well (Aggleton et al., 1986; Sapiurka et al., 2016). Thus, it is not possible to fully dissociate influences from each brain region when spatial working memory is tested via the T-maze. This is especially problematic when interpreting behavioral effects elicited during chronic stress regimens, where deficits in HPC function may precede changes in PFC (Cerqueira et al., 2007). The operant working memory task used in the present study was chosen because it depends specifically on the rodent mPFC and not the HPC (Sloan et al., 2006). Likewise, neurons of the mPFC are more robustly stimulated by exposure to varied stressors, whereas neurons of the HPC show a similar degree of stress-related neuronal activity regardless of whether the stressor is repeated or variable (Flak et al., 2012). As such, it is notable that the onset of working memory impairment in this operant task did not emerge until the third week of CVS, whereas impaired delayed alternation in the T-maze is apparent within the first week of repeated restraint (Hains et al., 2015). The comparatively delayed onset of stress-related impairment of PFC-dependent working memory may speak to a requirement for sustained exposure and progressively dysregulated neuroendocrine changes as CVS elicits HPA axis facilitation, whereas habituation is observed during repeated restraint stress (Flak et al., 2012; Radley and Sawchenko, 2015; Anderson et al., 2020).

Prefrontal cortex (PFC)-specific behaviors impaired by CVS, which facilitates HPA axis reactivity in young rats, are also useful to draw parallels with changes that emerge at advanced age, a time in the lifespan characterized by HPA axis dysregulation. Our present data affirm prior work detailing the age-dependent impairment of working memory in male rats, which is also dissociable from any concurrent decline in HPC-dependent behavior (Beas et al., 2013). Furthermore, individual differences in severity of age-related cognitive impairment correlate with HPA axis activity, but associations vary between cognitive domains. With respect to HPC-dependent cognition, aged individuals that exhibit compromised place-learning in the Morris water maze are also impaired in recovering GC levels to baseline after an acute stressor, relative to young adults or cognitively intact aged rats (Issa et al., 1990; Bizon et al., 2001). In relation to PFC-dependent cognition, aged individuals with less accurate working memory, as assessed on the operant DMTS task, show blunted GC secretion during acute stress while aged rats with more accurate working memory show enhanced GC secretion (McQuail et al., 2018). Independent of associations related to severity of either spatial learning or working memory are age-related increases to circadian GC secretion that constitute a continuous neuroendocrine challenge with which GR-expressing neurons must cope (Garrido et al., 2012). Such HPA axis dysregulation is practically poised to mediate age-related decline of PFC-dependent cognition as working memory is impaired in concert with CVS-related increases in mPFC corticosterone or following direct, intra-mPFC infusion of a GR-specific agonist (Roozendaal et al., 2004; Dominguez et al., 2019).

While our primary behavioral outcome was working memory choice accuracy, we also observed that normally aging rats or CVS-treated young adults completed fewer trials during the self-paced DMTS operant task compared to younger or unstressed controls. Fewer trials could reflect reduced motivation to respond for food rewards or greater sensitivity to reward devaluation as rats become sated during each test session. Normal aging is associated with lower motivation to work for food rewards in progressive ratio tasks (Hernandez et al., 2017; Jackson et al., 2021; Orsini et al., 2023) whereas pre-feeding to induce satiety and devalue food rewards is similar between young adult and aged rats (Samson et al., 2014). To the best of our knowledge, no studies have investigated the effect of CVS on either progressive ratio or outcome devaluation tasks. However, 21 days of repeated physical restraint decreases motivation of rats to respond for food across a range of reinforcement ratios (Kleen et al., 2006). Such changes in stressed rats are due to stress hormone signaling as either chronic oral corticosterone treatment or acute intra-cerebroventricular infusion of corticotropin releasing factor are sufficient to lower progressive ratio responding (Olausson et al., 2013; Bryce and Floresco, 2016). Importantly, and similar to aging, chronic stress, in the form of chronic social defeat, does not appear to affect reward devaluation by pre-feeding in mice (Dieterich et al., 2021). While it seems most parsimonious to conclude that age- or CVS-related reductions in trial completion during operant task performance are due to lower motivation to work to obtain food rewards, we caution that this secondary outcome requires further testing to determine if underlying mechanisms of aging and stress on motivation share overlapping neurobiological or physiological bases.

### 4.2 PFC molecular changes during stress or in advanced aging

Working memory is presumed to rely upon persistent activity within PFC cellular networks that encode representations of salient stimuli necessary to guide ongoing, goal-directed behavior (Goldman-Rakic, 1995). Persistent PFC neural firing and working memory are critically dependent on glutamatergic synaptic neurotransmission, chiefly via ionotropic NMDA receptors (Wang et al., 2013; McQuail et al., 2016; Auger and Floresco, 2017), but subtypes of mGluRs are also involved (Hernandez et al., 2018; Jin et al., 2018), as is GABAergic signaling (Wang et al., 2004; Bañuelos et al., 2014; Auger and Floresco, 2017). Furthermore, the rodent PFC is not a unitary structure, with evidence suggesting that effects of age or stress vary among its constituent subregions (Liston et al., 2006; Radley et al., 2006; Stranahan et al., 2012; Hernandez et al., 2018). Within this context, the second major finding of our study is that chronic stress increases mRNA expression of specific genes that encode for effectors of glutamatergic signaling in PrL whereas mRNA encoding for a transporter that modulates GABAergic tone is increased in the normally aging PrL. These effects of chronic stress or aging are specific to PrL as neither process reliably modulated glutamate or GABA gene expression in the IL.

Among mRNAs encoding for the essential and major accessory NMDAR subunits that were assessed in our study, expression of *Grin2b*, which encodes for the GluN2B protein, was significantly greater in the PrL after 21 days of CVS without any corresponding change in the IL. The expression of *Grin2b* is consequential because NMDARs are enriched at thin spines (Kasai et al., 2003; Noguchi et al., 2005) and activation of GluN2B-containing NMDARs is essential to induce cortical synaptic plasticity, including in the PFC (Cui et al., 2011; Plattner et al., 2014). While chronic stress provokes changes to mPFC neuronal morphology and synaptic function in an NMDAR-dependent manner (Li et al., 2011; Martin and Wellman, 2011), there is mixed evidence as to how NMDARs themselves are regulated by chronic stress in the PFC. Postmortem analyses of PFC tissues obtained from patients diagnosed with major depressive disorder (MDD) have variously reported that GluN2B protein may be decreased (Feyissa et al., 2009) or increased (Gray et al., 2015). Likewise, studies of chronic stress in aging rodents vary in the observation of lower (Lee and Goto, 2011; Yuen et al., 2012; Shepard and Coutellier, 2018) or greater levels of *Grin2b*/GluN2B (Erburu et al., 2017; Li et al., 2018; Zhu et al., 2021). The origin of these discrepancies is debatable, but we note that studies reporting loss of GluN2B examined expression after 7–14 days of chronic stress whereas those reporting increased GluN2B did so after 21–28 days of stress. Indeed, the timeline of chronic stress effects on GluN2B are multiphasic; there is an initial increase of GluN2B at the cell surface membrane on the first day of stress that is followed by lower levels through Day 7 and this loss is reversed after 5 days of post-stress recovery (Yuen et al., 2012). While changes have not been systematically studied in relation to longer durations of stress, the present observation of greater *Grin2b* after 21 days of CVS fits the direction and timing of Erburu et al. (2017), Li et al. (2018), and Zhu et al. (2021), and may reflect continuing oscillation of *Grin2b* expression during sustained exposure to stressors. However, this increase is not likely to persist indefinitely as *Grin2b* in PFC of chronically stressed rats is no longer different from unstressed controls after 42 days (Calabrese et al., 2012).

We also observed that the CVS-dependent increase in *Grin2b* was contemporaneous with increases in expression of *Grm4* and *Plcb1*, other genes that encode proteins associated with regulation of glutamatergic signaling. *Grm4* codes for mGluR4 which, like other Group III mGluRs, inhibits adenylate cyclase and synaptic glutamate release (Conn and Pin, 1997). Relative to NMDARs, fewer studies have implicated or investigated contributions of *Grm4* expression or mGluR4 signaling to the pathophysiology of stress-related disorders that impact the PFC. Gray et al. (2015) reported increased *Grm4* in the PFC of patients diagnosed with MDD whereas concentration of cinnabarinic acid, a kynurenine metabolite and agonist of mGluR4, is lower in the PFC of schizophrenic patients (Ulivieri et al., 2020). *Grm4* may be targeted by chronic stress in the PFC as Wan et al. (2018) also reported stress-related increase of *Grm4* in the rat PFC. *Plcb1* encodes for phospholipase C beta 1 (PLCβ1), an enzyme linked with post-synaptic mGluR signaling (Conn and Pin, 1997). PLCβ1 transduces signaling that originates from Group I mGluRs, comprised of mGluR1 and mGluR5. Signaling via mGluR5 in the PrL is required for optimal working memory (Hernandez et al., 2018), an effect which may pertain to mGluR5-mediated enhancement of NMDAR signaling and which contrasts with mixed effects of mGluR1 activation (Mannaioni et al., 2001). Though the expression of *Grm5* or *Grm1* was similar between treatment groups in our CVS study, others have reported loss of mGluR5 protein from the PFC in MDD patients (Deschwanden et al., 2011; Jernigan et al., 2011; Kim et al., 2019). Therefore, our observation of coordinated, upregulation of *Grin2b*, *Grm4*, and *Plcb1* in the stressed PrL may reflect molecular remodeling of glutamatergic synapses that support working memory, although further studies at the levels of protein expression, function, and physiology are needed to confirm. Furthermore, we also acknowledge that neurobiological effects may vary due to differences in stress paradigm or duration (Yuen et al., 2009, 2012; Flak et al., 2012) and that factors beyond gene transcription certainly determine the functional consequences of expressed gene products to mPFC synapse physiology, including protein trafficking (Erburu et al., 2017), phosphorylation (Li et al., 2018), and degradation (Yuen et al., 2012).

When identical molecular methods were applied to study age-related changes, we found that aging significantly increased expression of *Slc6a11*, which encodes for GABA transporter type 3 (GAT-3), in the PrL. Several studies have highlighted a mechanistic role for mPFC inhibition in the age-related decline of working memory (McQuail et al., 2015). Tonic inhibition, chiefly transduced via extra-synaptic GABABRs, increases with age and couples with individual differences in severity of working memory impairment (McQuail et al., 2012; Bañuelos et al., 2014; Carpenter et al., 2016). When excess tonic inhibition is blocked in the aged mPFC, working memory is restored (Bañuelos et al., 2014). The origin of increased extracellular GABA in the aging mPFC may be ascribed to deficient GABA transport as level of the predominant neuronal GABA transporter, GAT-1, declines with age (Bañuelos et al., 2014). GAT-3, found on astrocytes, differs from GAT-1 in its ability to release GABA, even under normal physiological circumstances, and regulates activity of GABABRs (Kinney, 2005; Ueberbach et al., 2023). It is not clear whether increased *Slc6a11* expression contributes meaningfully to mPFC tonic inhibition and impaired working memory in aging. This is due to the fact that any alteration to astrocytic regulation of extracellular GABA would likely interact with concurrent changes to neuronal sources of GABA that include, on the one hand, fewer interneurons containing the 67-kDa isoform of glutamic acid decarboxylase (GAD-67; Stranahan et al., 2012) but, on the other hand, an overall increase in GAD-67 protein level (Bañuelos et al., 2014). However, these new molecular data complement existing biochemical and physiological evidence that implicates enhanced GABAergic inhibition as a consistent signature of normal PFC aging and a likely contributor to suppression of cellular activity required for optimal working memory.

### 4.3 Global PFC glutamate and GABA profiles in stress and advanced aging

This study leveraged a PCR array approach to investigate 55 genes associated with GABA and glutamate signaling in PrL or IL of rats that were either exposed to CVS or allowed to age normally. To compare the molecular profiles of CVS with normal aging, changes in gene expression (Log2FC) relative to the control group (unstressed, young adult) for all glutamate and GABA-related genes of interest were tested for association in each mPFC subregion. As such, the third finding of our study is that gene expression patterns from the CVS and aging experiments are positively correlated in the PrL dataset but not correlated in the IL dataset. This observation provides evidence for a broader, shared pattern of glutamate and GABA gene regulation between chronic stress or normal aging at the level of PrL. The subregion-specificity of this association is not surprising given chronic stress is known to produce divergent changes in dendritic morphology across PFC subregions (e.g., anterior cingulate vs. orbitofrontal; Liston et al., 2006) so the present molecular data further localize effects on gene expression that vary between subregions of the mPFC. Likewise, others have reported subregion-specific changes to cellular and molecular profiles between mPFC subregions during normal aging (Stranahan et al., 2012; Hernandez et al., 2018). So, even if the specific glutamate- and GABA-related genes that are differentially expressed during chronic stress or with age are not identical, our data support that future studies must parse effects by mPFC subregion and that a focus on PrL may be especially informative when seeking to delineate GC-mediated processes that impair working memory in the contexts of chronic stress or age-related HPA axis dysfunction.

### 4.4 Limitations and considerations

We implemented parallel studies of working memory and PFC gene expression to identify potential overlap of behavioral and molecular phenotypes in advanced aging or after chronic variable stress as both are associated with HPA axis dysregulation. While the findings discussed here are consistent with some predictions of the GC hypothesis, there are limitations that should be considered. First, aberrant GC signaling is one process among several proposed to drive brain aging and neurodegeneration; other candidate processes include, but are not limited to, inflammation, protein aggregation, bioenergetic imbalance, and DNA damage (Wilson et al., 2023). Indeed, relevant processes are not mutually exclusive, and their relative impacts may vary in shaping cognitive and neural outcomes across the lifespan. Critically, our present study is descriptive and did not examine causal relations between chronic stress or GC signaling and PFC-dependent working memory or gene expression in rodents of advanced age. Future experiments can build on these data by examining the effects of CVS in aging rats in either a cross-sectional or longitudinal fashion and reveal the role for GC signaling using pharmacological or molecular tools to modulate the formation of GCs or actions of corticosteroid receptors in the aging brain. Second, parallel behavioral and molecular procedures facilitated the comparison of normal aging and CVS phenotypes on working memory and PFC gene expression, but practical considerations prevented the creation of fully matched conditions between cohorts of aged or chronically stressed rats. Owing in part to limitations in availability of aging rats from the National Institute on Aging, the same young adult and aged rats were used for both behavioral testing and gene expression studies, albeit with a two-week recovery interval to separate behavioral testing from tissue collection. However, it was not possible to use the same cohort of CVS rats to assess both behavior and PFC gene expression, as we could not allow for 14 days of recovery from CVS. As such, we prepared a second cohort of young adult rats for dedicated analysis of CVS effects on PFC gene expression, separate from those rats used to delineate the time-course of CVS effects on working memory. Significantly, this means that gene expression was examined in a normally aging cohort with a history of behavioral testing and in a CVS cohort without any history of behavioral testing. We partially corrected for this in our comparative analysis of gene expression by first normalizing to the appropriate internal control groups (unstressed, young adult rats with history of behavioral testing in the aging cohort or unstressed, young adult rats without history of behavioral testing in the CVS cohort) before conducting the comparative analysis across cohorts. However, it is important to emphasize that the influence of behavioral history is not fully controlled for, even with those intra-study corrections.

## 5 Conclusion

The GC hypothesis proposes that cumulative effects of stress hormones acting upon sensitive neuronal populations contribute to the process of brain aging. Evidence for this hypothesis is apparent as dysfunction of the HPA axis in dementia patients but also heightened risk for dementia among individuals with a greater history of adverse experiences or stressful exposures across the lifespan (Hartmann et al., 1997; Wilson et al., 2005, 2011; Csernansky et al., 2006; Ennis et al., 2017; Majoka and Schimming, 2021). Controlled studies in normally aging preclinical models affirm connections between HPA axis dysregulation and memory loss, especially in relation to HPC-dependent mnemonic processes (Issa et al., 1990; Morano et al., 1994; Yau et al., 1995; Bizon et al., 2001). Our new data demonstrate that PFC-dependent working memory is commonly susceptible to decline in advanced aging or after chronic variable stress and these impairments coincide with changes to glutamate and GABA gene expression within the PrL subregion of the rodent mPFC. The overlap of behavioral and molecular phenotypes generally extends the application of the GC hypothesis to PFC aging at the levels of working memory and PFC glutamate and GABA gene expression. However, differentially expressed genes of the stressed PrL may be contrasted with those changed with age in the PrL; the former reflects changes to NMDAR and mGluR signaling, and the latter changes to extracellular GABA. These differences may explain, in part, why PFC neural responses to stressful stimuli vary with advancing age (Lowy et al., 1995; Bloss et al., 2010) and draw attention to the need for further studies that examine interactions between stress and aging. These could include, on the one hand, examining the delayed or longitudinal effects of stress on trajectories of age-related working memory decline, and on the other, the effects of chronic stress on working memory that is changing across the full lifespan. Such studies would be foundational to understand the mechanisms by which stress can increase risk for cognitive impairment at older ages and how the aging PFC supports cognition during stress.

## Data availability statement

The original contributions presented in this study are included in this article/supplementary materials, further inquiries can be directed to the corresponding author.

## Ethics statement

The animal study was approved by the University of Florida Institutional Animal Care and Use Committee. The study was conducted in accordance with the local legislation and institutional requirements.

## Author contributions

HG: Formal analysis, Investigation, Visualization, Writing – original draft, Writing – review & editing. FH: Writing – review & editing. CH: Formal analysis, Investigation, Writing – review & editing. JM: Conceptualization, Formal analysis, Funding acquisition, Investigation, Project administration, Supervision, Visualization, Writing – original draft, Writing – review & editing.
